# Genome Sequences of Five Arenaviruses from Pygmy Mice (Mus minutoides) in Sierra Leone

**DOI:** 10.1128/mra.00095-22

**Published:** 2022-04-07

**Authors:** Matej Vučak, James Bangura, Bruno M. Ghersi, Jenna Nichols, Joseph Hughes, Ana da Silva Filipe, Alexandre Tremeau-Bravard, David J. Wolking, Emmanuel Amara, Abdulai Bangura, Marilyn C. Kanu, Osman T. Kanu, Dickson Kargbo, Edwin G. Lavalie, Victor Lungay, Willie Robert, Mohamed Turay, Steven Fornie, Thomas T. Samba, Bankolay B. Sesay, Patrick Swaray, Mohamed A. Vandi, Mohamed Alpha Bah, Andrew A. Mansaray, Brian H. Bird, Andrew J. Davison

**Affiliations:** a MRC-University of Glasgow Centre for Virus Research, Glasgow, United Kingdom; b University of Makeni and University of California, Davis One Health Program, Makeni, Sierra Leone; c One Health Institute, School of Veterinary Medicine, University of California, Davis, Davis, California, USA; d Ministry of Health and Sanitation, Freetown, Sierra Leone; e Ministry of Agriculture and Forestry, Freetown, Sierra Leone; KU Leuven

## Abstract

The genome sequences of five strains of a mammarenavirus were assembled from metagenomic data from pygmy mice (Mus minutoides) captured in Sierra Leone. The nearest fully sequenced relatives of this virus, which was named Seli virus, are lymphocytic choriomeningitis virus, Lunk virus, and Ryukyu virus.

## ANNOUNCEMENT

Members of genus *Mammarenavirus* in family *Arenaviridae* have genomes consisting of two single-stranded ambisense RNA segments (L and S) and most have rodents as hosts (1). Among a collection of rodents captured in Sierra Leone that had tested positive for the mammarenavirus Lassa virus (LASV), we used visual recognition, DNA barcoding of the *MT-CO1* gene (2), and sequencing of the *MT-CO1* and *MT-CYB* genes to identify five apparently healthy animals from Koinadugu District as pygmy mice (Mus minutoides), in which LASV has been reported occasionally (3). An additional 18 pygmy mice in this collection were LASV negative. The five mice had tested positive for LASV in two assays for the L gene (encoding the RNA-directed RNA polymerase) but not in an assay for the GPC gene (encoding the glycoprotein precursor) (Table 1, see footnotes b and c for assay details). We determined the genome sequences of the mammarenaviruses in these mice.

**TABLE 1:** Data on SELV in this study^*a*^

Animal	Location^*b*^	qPCR LASV load by sample type (*C_T_*)				Size (nt) of:		G+C (%) content of:		NCBI SRA accession no. by sample type			NCBI GenBank accession no. of:			
		BL^*c*^	OS^*c*^	US^*c*^	BL^*d*^	L^*e*^	S^*e*^	L	S	BL	OS	US	L	S	*MT-CO1* ^*f*^	*MT-CYB* ^*f*^
00094	Sengbeh, Gbenikoro	31.7	36.4	ND	27.8	7,173 (7,160)	3,390 (3,377)	45	47	SRX13144664	SRX13144665	SRX13144671	OL364850	OL364851	OL415139	OL441084
00127	Wara Wara Bafodia, Bafodia	38.6	ND	ND	27.2	7,210 (7,171)	3,389 (3,384)	44	47	SRX13144672	SRX13144673	SRX13144674	OL364852	OL364853	OL415140	OL441085
00207	Diang, Badala	27.5	31.7	37.6	21.7	7,180 (7,180)	3,389 (3,384)	44	47	SRX13144675	SRX13144676	SRX13144677	OL364854	OL364855	OL415141	OL441086
00367	Wara Wara Bafodia, Bafodia	31.6	32.3	31.6	27.4	7,173 (7,173)	3,389 (3,389)	45	47	SRX13144678	SRX13144666	SRX13144667	OL364856	OL364857	OL415142	OL441087
00414	Diang, Badala	28.6	28.9	35.7	21.5	7,181 (7,176)	3,388 (3,363)	45	47	SRX13144668	SRX13144669	SRX13144670	OL364858	OL364859	OL415143	OL441088

a NCBI BioProject no. PRJNA780418. *C_T_*, cycle threshold; BL, blood sample; OS, oral swab sample; US, urogenital swab sample; ND, not detected; nt, nucleotide; L, L segment; S, S segment; SRA, sequence read archive; *MT-CO1*, mitochondrial gene encoding cytochrome c oxidase I; *MT-CYB*, mitochondrial gene encoding cytochrome b.

b Chiefdom and site in Koinadugu District.

c Using a method targeting the L gene (16).

d Using a RealStar LASV RT-PCR 2.0 kit targeting the L gene (Altona Diagnostics). LASV was not detected in these samples using a RealStar LASV RT-PCR 2.0 kit targeting the GPC gene.

e Including undetermined nucleotides at the segment termini. The size without these nucleotides is given in parentheses.

*^f^* Sequence determined from the combined data set for each animal and by Illumina sequencing of OS DNA from each animal.

Blood, oral swab, and urogenital swab samples were obtained from each mouse. Nucleic acids were extracted using Applied Biosystems MagMAX CORE and pathogen kits with a KingFisher Duo Prime purification system and treated with Ambion DNase I. RNA was purified using the Beckman Coulter RNAClean XP kit and depleted of rRNA using an Illumina Ribo-Zero Plus kit. A metagenomic method (G-Meta) (4) was used to prepare cDNA and sequencing libraries, which were analyzed on an Illumina NextSeq 550 instrument using a high-output 300-cycle cartridge.

Bioinformatic tools were used with default parameters unless specified otherwise. The genome sequences were determined using a pipeline built around SPAdes v3.13 (5) and DIAMOND v2.0.9 (6) (https://github.com/mvvucak/LASV_Sample_Classification/). The 15 data sets (each consisting of 5,076,546 to 34,278,974 paired-end reads) were quality filtered using Trim Galore v0.4.0 (https://www.bioinformatics.babraham.ac.uk/projects/trim_galore/). The three data sets for each mouse were combined and assembled *de novo*, and contigs related to mammarenavirus sequences were identified. The contigs were joined into genome sequences manually by iteratively incorporating reads sharing short sequences near their ends. Individual and combined data sets were then mapped to the genome sequences using Bowtie 2 v2.3.1 (7) with the ‐‐local parameter. The alignments were visualized using Tablet v1.21.02.08 (8), and final adjustments to the consensus sequences were made. Published information on other mammarenaviruses was used to locate the segment ends when possible and to annotate the genome sequences.

**FIG 1:**
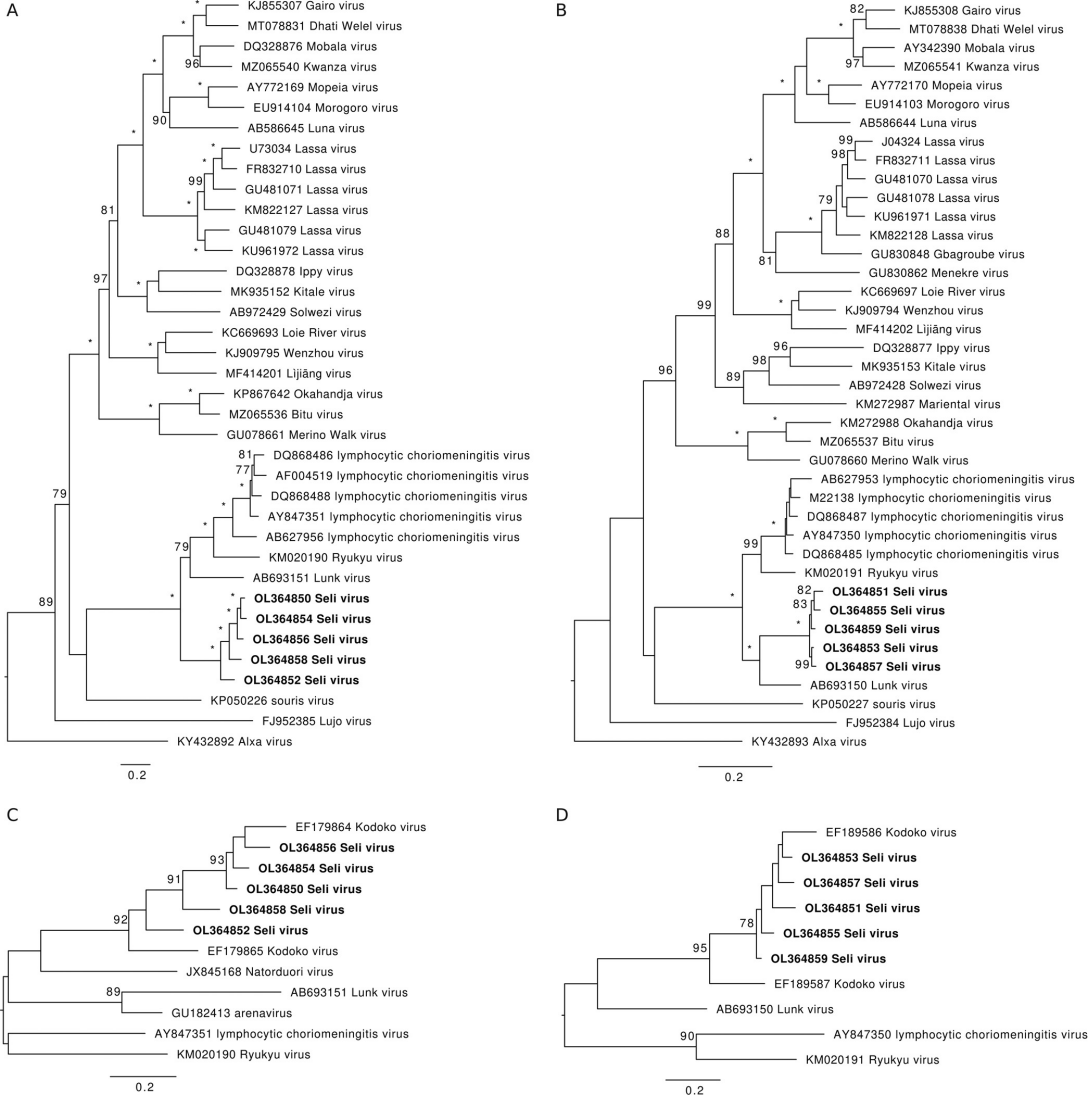
Phylogenetic analyses of SELV. The complete amino acid sequences of the L (A) and the NP (B) proteins of Old World mammarenaviruses and partial nucleotide sequences of the L (C) and the NP (D) genes of SELV and its closer relatives were aligned using MAFFT v7.475 (13). The alignments were inspected visually in JalView v2.11 (14). Maximum likelihood phylogenies for each alignment were reconstructed using IQ-Tree v2.1.3 (15) by instituting the best fitting model selected by ModelFinder according to the Bayesian information criterion. The trees in A and B were rooted using a New World mammarenavirus, Junín virus (not shown; GenBank accession no. AY358022 and AY358023, respectively). The trees in C and D were rooted using Lassa virus (not shown; GenBank accession no. U73034 and J04324, respectively). Branch support was assessed using 1,000 bootstrap replicates, with values above 75% shown at the nodes and values of 100% indicated by asterisks. The viruses are denoted by GenBank accession no. and name, and SELV strains are indicated by bold font. The scale bars represent substitutions per site.

The analysis indicated that a single virus, in some instances with a few single-nucleotide variants, was present in each mouse. Phylogenetic analyses showed that these viruses represent strains of a mammarenavirus, which we have named Seli virus (SELV) after a river in Sierra Leone (Fig. 1). Thus, among fully sequenced mammarenaviruses, SELV is most closely related to Lunk virus, which was isolated from a pygmy mouse in Zambia (9); Ryukyu virus, which was isolated from a Ryukyu mouse (Mus caroli) in China (10); and lymphocytic choriomeningitis virus, which is widespread in house mice (Mus musculus) (1) (Fig. 1A and B). SELV is sufficiently distinct to meet the formal criteria for assignment to a new viral species (1), to which additional phylogenetic analyses based on short sequences suggest that Kodoko virus, from pygmy mice in Guinea (11), may also belong (Fig. 1C and D). Further mapping experiments using Bowtie 2 as described above showed that reads originating from recognized LASV clades (12) were completely absent from all 15 data sets. This finding suggests that the L gene assays used are capable of detecting SELV as well as LASV.

The work was conducted in partnership with the Government of Sierra Leone Ministry of Agriculture and Forestry representatives working under a ministry-approved wildlife specimen collection permit, and with further approvals from the University of California, Davis Institutional Animal Care and Use Committee.

### Data availability.

Sequence information and accession numbers are provided in Table 1.
